# The elusive relationship between cardiac filling and fluid responsiveness

**DOI:** 10.1186/s13054-024-04861-y

**Published:** 2024-03-15

**Authors:** Jon-Emile S. Kenny, Ross Prager, Korbin Haycock

**Affiliations:** 1 Health Sciences North Research Institute, 56 Walford Rd, Sudbury, ON P3E 2H2 Canada; 2 Flosonics Medical, Toronto, ON Canada; 3 Division of Critical Care Medicine, Western University, London, ON Canada; 4 Department of Emergency Medicine, Riverside University Health System Medical Center, Moreno Valley, CA USA

## Editor:

Munoz et al. [1] have recently published an important pilot study in *Critical Care*. In 90 mechanically ventilated patients with circulatory failure who were admitted to the intensive care unit (ICU) for less than 24 h, four measures of ‘venous congestion’ (VC) were related to fluid responsiveness (*F*_R_) or unresponsiveness (*F*_UR_). To score VC, each patient received 1 point for any of the following: (1) a central venous pressure (CVP) > 12 mmHg; (2) a lung ultrasound score (LUS) > 10; (3) a venous excess ultrasound score (VExUS) > 1; and (4) a lateral *E*/*e*’ > 10 for the left ventricle. Additionally, *F*_R_ was measured pragmatically based on either pulse pressure variation, stroke volume variation, passive leg raising, or end expiratory occlusion test (considering the presence of arrhythmias, spontaneous ventilation, and the availability of monitoring devices). The authors hypothesized that F_R_ patients would have fewer VC signals than F_UR_ patients. Contrary to these expectations, there was no statistically significant difference between F_R_ and F_UR_ patients with respect to number of VC signals. Fifty-three percent of F_R_ patients had at least one VC signal; only slightly more (57%) of F_UR_ showed at least one VC signal. From this, the authors correctly concluded that VC is present in a clinically significant fraction of *F*_R_ patients and clinicians should, therefore, be wary when resuscitating based only upon measures of *F*_R_. However, we note that their results are equally true in the reverse sense which brings to mind the fundamental relationship between surrogates of cardiac filling (e.g., VC or lack thereof) and the response to additional preload (i.e., *F*_R_). For example, the frequently used strategy of prescribing fluids based on a lower CVP or flat IVC [2].

Though not explicitly mentioned by Munoz and colleagues, of equal importance in their data is the high fraction of patients without VC (i.e., normal venous measures) who were, nonetheless, *F*_UR_. Specifically, 43% of patients with *normal venous measures* were predicted to *not* increase stroke volume with another fluid bolus. The takeaway from this observation is that deciding to give IV fluid based only upon venous measures is also problematic. This is even more striking when looking only at VExUS score, which all 90 patients had calculated (supplemental data). For those patients who were *F*_R_, approximately 97% had a VExUS score of zero or 1. However, roughly 87% of patients who were F_UR_ also had a VExUS score of zero or 1; thus, the specificity of a VExUS of zero or 1 for detecting *F*_R_ is only 13%!

**Fig. 1:**
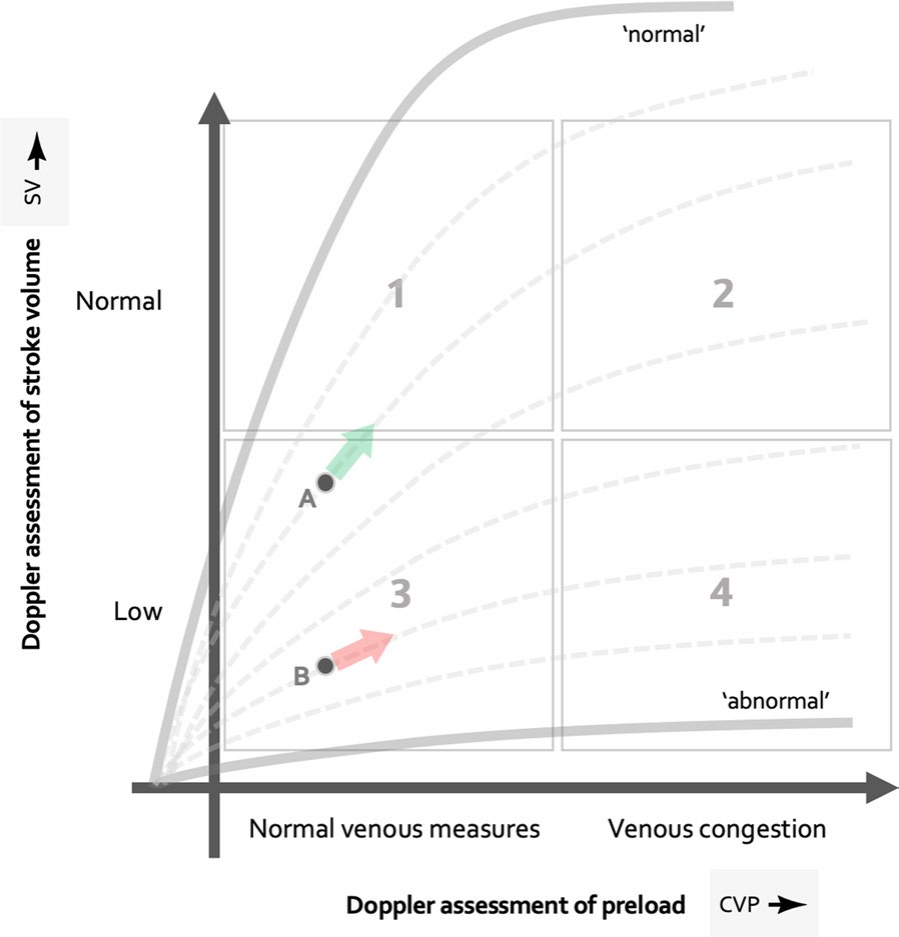
The Doppler starling curve. The four hemodynamic phenotypes (1–4) are generated by combinations of normal and low stroke volume on the *y*-axis and normal or congested venous measures on the *x*-axis. The family of Starling curves (dotted lines) shows that a patient can have normal venous measures and be responsive (patient A, green) or unresponsive (patient B, red). If a population is studied with cardiac dysfunction, more patient B physiology will be observed

How do we make sense of these findings? One approach is to return to basic cardiac physiology and consider the shape of the Frank–Starling curve as it relates cardiac filling on the *x*-axis and stroke volume on the *y*-axis (see Fig. 1) [3–5]. If the entire population of patients had normal, upright cardiac function curves that flatten out at higher filling pressure, then normal/low venous measures would associate with *F*_R_ (i.e., ascending curve) and VC with *F*_UR_ (i.e., flat curve), as hypothesized by Munoz and colleagues. However, if greater numbers of patients with flattened cardiac function curves are studied, then being F_UR_ and having normal venous measures will necessarily be more common. We have recently described this physiology in terms of a ‘Doppler Starling curve’ where normal venous measures (i.e., VExUS zero or 1) and *F*_UR_ may be observed in hemodynamic ‘quadrants’ (*Q*_*x*_) *Q*_1_ and *Q*_3_ (and theoretically more common in *Q*_3_ than *Q*_1_, see Fig. 1). In a pilot study using simultaneous common carotid artery and internal jugular Doppler ultrasonography in emergency department (ED) patients deemed to require intravenous fluid expansion we, like Munoz and colleagues, observed a high proportion (i.e., 67%) of F_UR_ assessments with jugular venous Doppler morphologies consistent with low preload (i.e., normal venous measures) [4]. On the other hand, like Munoz et al., we observed that a clinically significant (i.e., 14%) fraction of *F*_R_ assessments occurred when there was a pulsatile, congested jugular venous Doppler morphology. Within the Doppler Starling curve framework, this constellation is seen in *Q*_2_ and *Q*_4_ with the proportion determined by the severity of cardiac dysfunction in the population studied (see Fig. 1). Taken together, the poor relationship between venous measures and the presence or absence of *F*_R_ speaks to the diversity of cardiac function curves in the ICU. Importantly, neither ejection fraction [6], nor LV fractional shortening (as observed by the authors) can accurately determine the slope of the Frank–Starling curve—it demands a dynamic assessment! Deciding IV fluids based only on *F*_R_/*F*_UR_ or based only on venous measures (e.g., VExUS) carries equal risk—both the *x*- and *y*-axes of the Frank–Starling relationship should be serially measured during resuscitation to best tailor IV fluid therapy.

In summary, Munoz and colleagues have published an influential pilot investigation, executed with rigor. We should continue to anticipate little relation between venous measures and fluid responsiveness, especially with impaired cardiac function. Doppler phenotyping could be useful to better delineate the safety profile of IV fluids throughout the course of illness.

## Data Availability

Not applicable.

## References

[1] Muñoz F, Born P, Bruna M, Ulloa R, González C, Philp V, Mondaca R, Blanco JP, Valenzuela ED, Retamal J (2024). Coexistence of a fluid responsive state and venous congestion signals in critically ill patients: a multicenter observational proof-of-concept study. Crit Care.

[2] Millington SJ, Koenig S (2021). Ultrasound assessment of the inferior vena cava for fluid responsiveness: making the case for skepticism. J Intensive Care Med.

[3] Kenny J-ES (2022). Assessing fluid intolerance with Doppler ultrasonography: a physiological framework. Med Sci.

[4] Kenny J-ÉS, Prager R, Rola P, Haycock K, Gibbs SO, Johnston DH, Horner C, Eibl JK, Lau VC, Kemp BO. Simultaneous venous-arterial Doppler ultrasound during early fluid resuscitation to characterize a novel Doppler starling curve: a prospective observational pilot study. J Intensive Care Med 2024:08850666231224396. 10.1177/08850666231224396 PMC11188059 38190576

[5] Kenny JS, Prager R, Rola P, Haycock K, Basmaji J, Hernández G (2023). Unifying fluid responsiveness and tolerance with physiology: a dynamic interpretation of the diamond-forrester classification. Crit Care Explor.

[6] Mahjoub Y, Benoit-Fallet H, Airapetian N, Lorne E, Levrard M, Seydi A-A, Amennouche N, Slama M, Dupont H (2012). Improvement of left ventricular relaxation as assessed by tissue Doppler imaging in fluid-responsive critically ill septic patients. Intensive Care Med.

